# Supplementary material for: Cathode engineering with perylene-diimide interlayer enabling over 17% efficiency single-junction organic solar cells
Source: Nat Commun. 2020 Jun 1;11:2726. doi: 10.1038/s41467-020-16509-w (PMC7264349; doi:10.1038/s41467-020-16509-w)
Supplement: Supplementary file 1 — Supplementary Information [file 41467_2020_16509_MOESM1_ESM.pdf]

Cathode Engineering with Perylene-diimide Interlayer Enabling Over 17% Efficiency Single-junction Organic Solar Cells

Yao *et al.*

SUPPLEMENTARY FIGURES

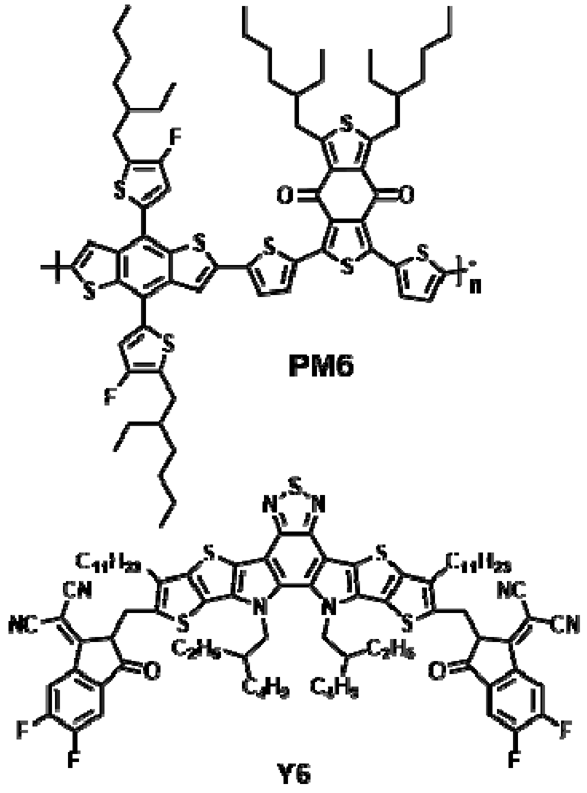

Supplementary Figure 1. Molecule structures of PM6 donor and Y6 acceptor.

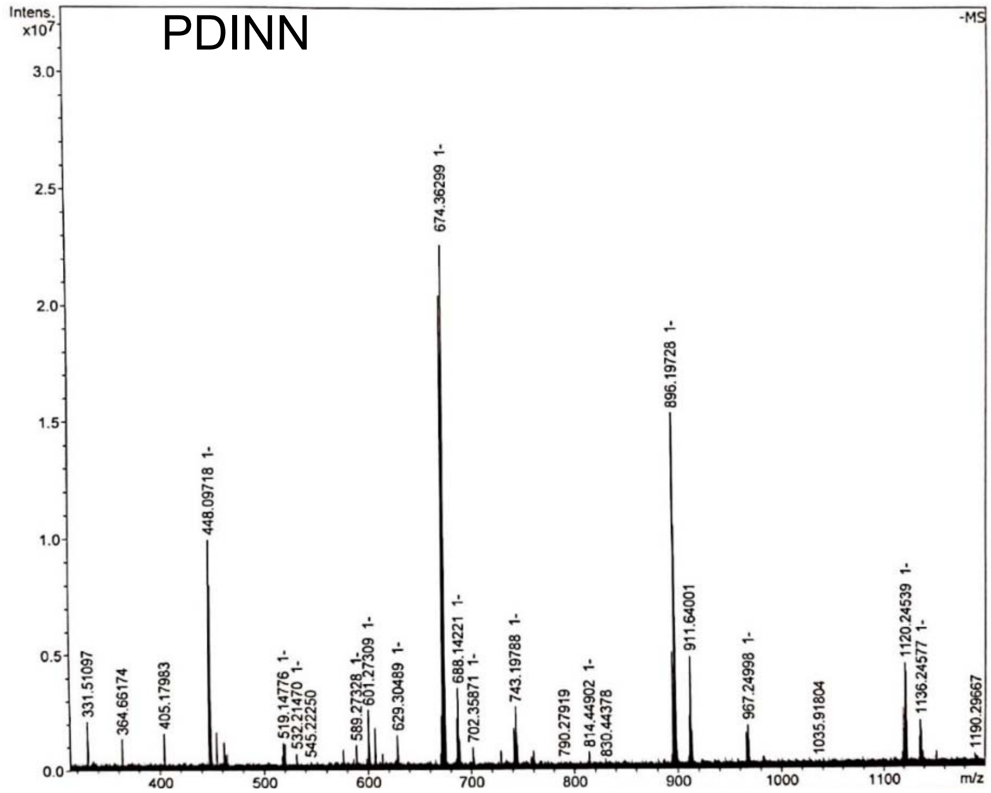

Supplementary Figure 2. MALDI-TOF mass spectrometry of PDINN.

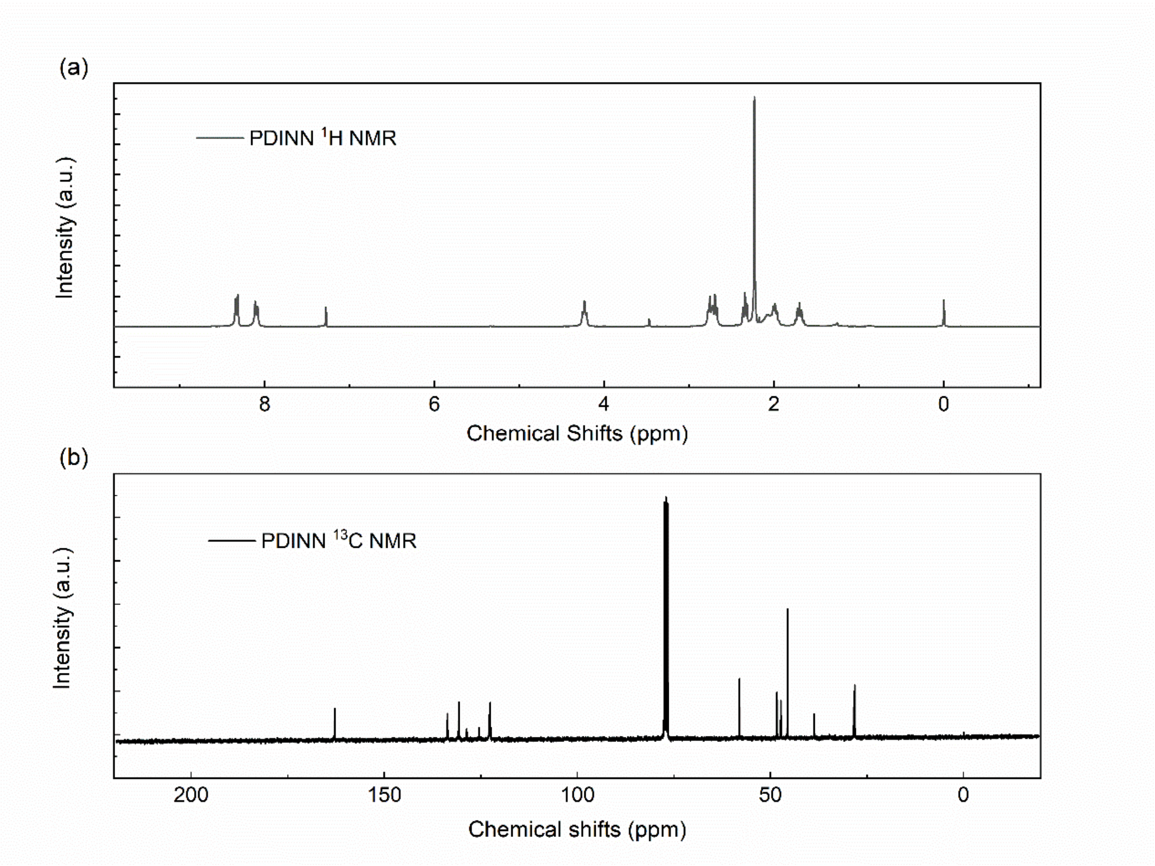

Supplementary Figure 3. (a) ^{1}H NMR and (b) ^{13}C NMR spectra of PDINN.

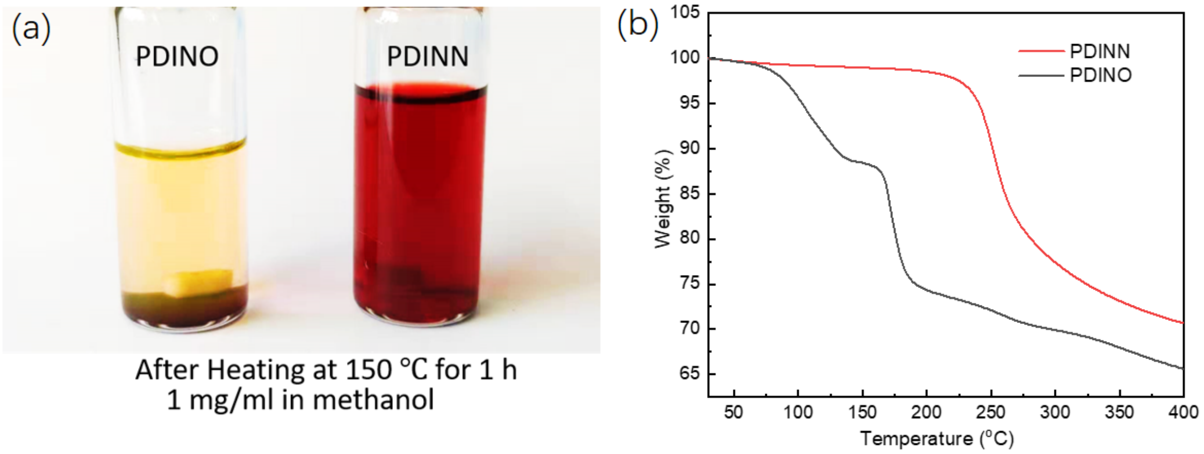

Supplementary Figure 4. (a) Solubility of heated PDINO, PDINN in methanol. (PDINO and PDINN powder is heated before dissolving with methanol). (b) TGA plots of PDINN and PDINO.

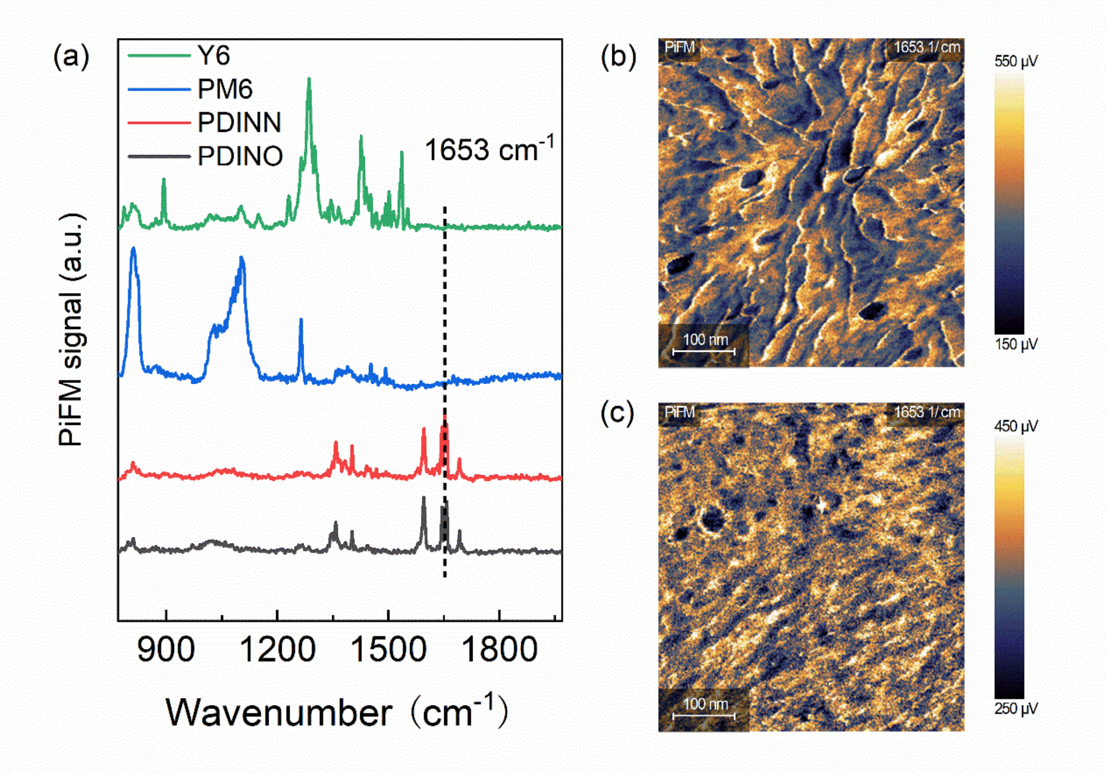

Supplementary Figure 5. (a) Infrared (IR) spectra of PM6, Y6, PDINN and PDINO neat films and PiFM images of ultrathin (b) PDINO and (c) PDINN films which were deposited on PM6:Y6 active layer.

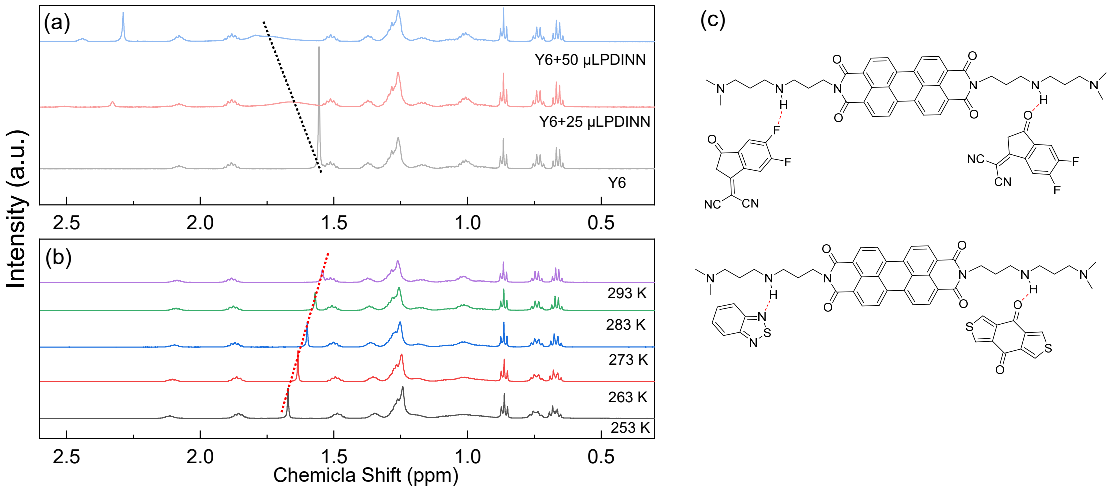

Supplementary Figure 6. (a, b) Variable-temperature and titration ^{1}H NMR experiments conducted with PDINN and Y6 in solution; (c) Proposed model for the hydrogen-bond formation for PDINN.

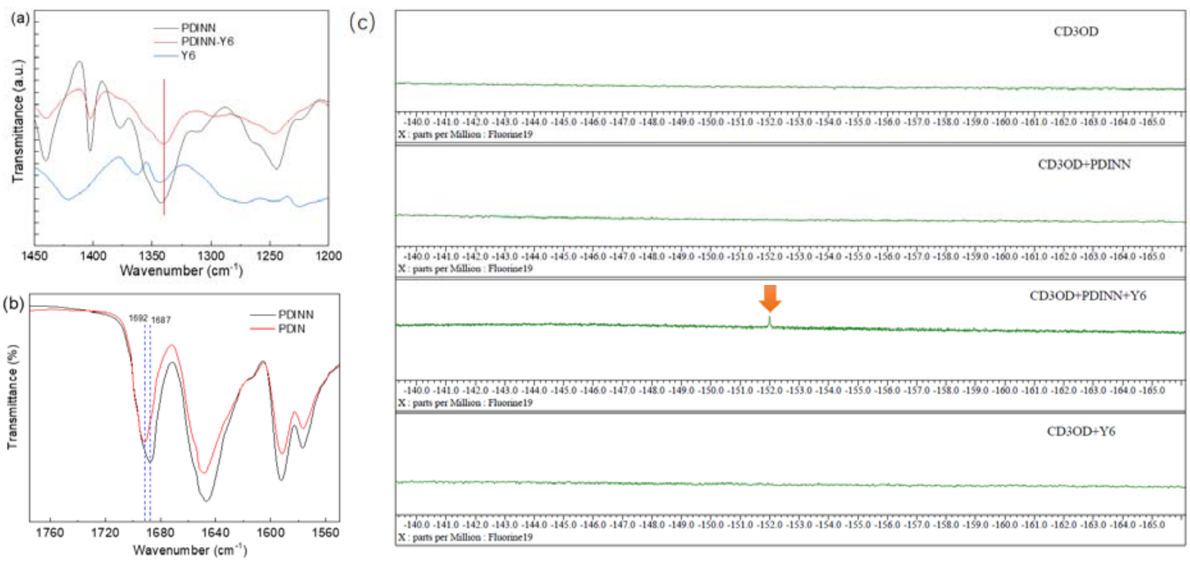

Supplementary Figure 7. (a) The ATR FT-IR spectra of Y6 and PDINN/Y6 (w/w, 1:1) blend, (b) the ^{19}F NMR spectra. (c) The ATR FT-IR spectra of PDIN and PDINN.

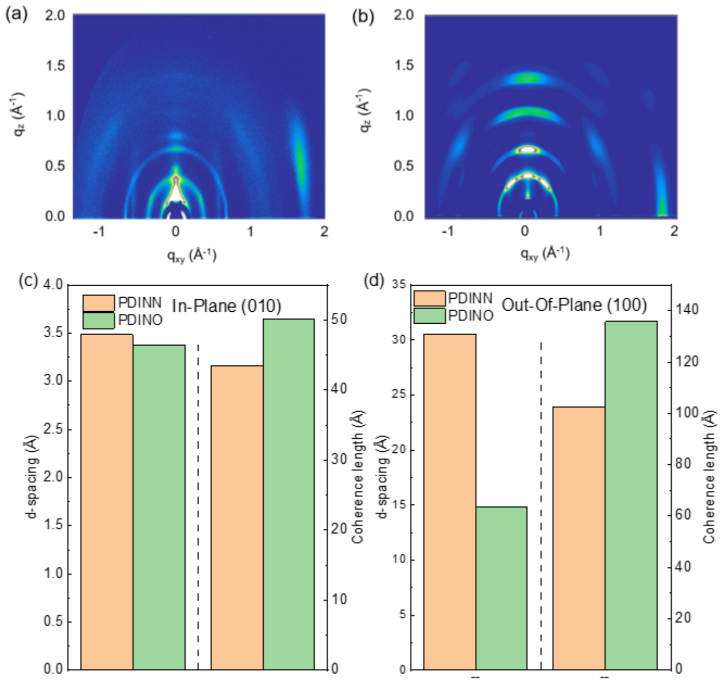

Supplementary Figure 8. Grazing-incidence wide-angle X-ray scattering (GIWAXS) pattern of (a) PDINN and (b) PDINO. The d-spacing and coherence length estimated from the (c) in-plane (010) and (d) out-of-plane (100) diffraction of PDINO and PDINN.

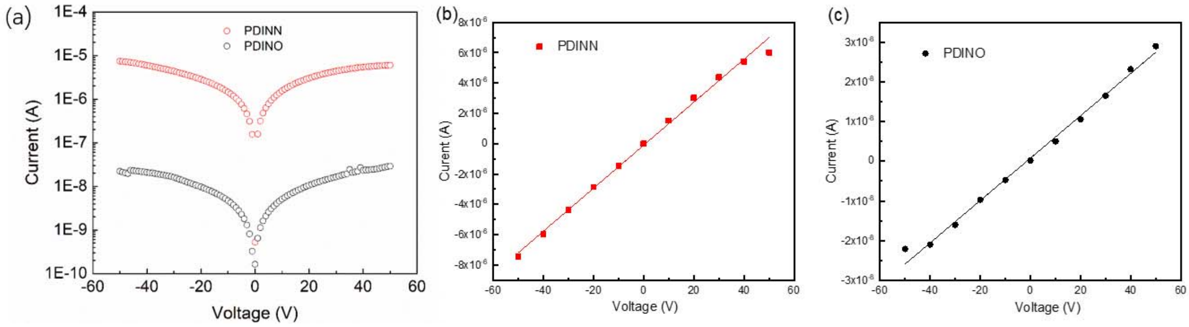

Supplementary Figure 9. (a) Current-voltage (*I*-*V*) measurements of PDINO and PDINN thin films. The corresponding data are plotted on linear-linear scale for (b) PDINN and (c) PDINO.

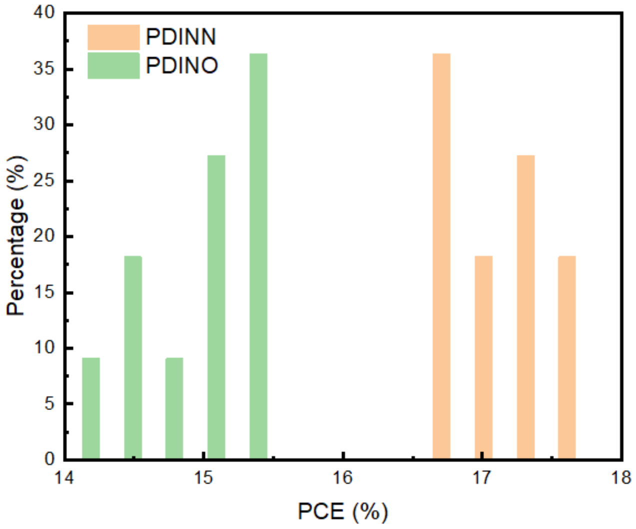

Supplementary Figure 10. The efficiency histograms of the PDINN-based and PDINO-based OSCs.

page 7

ICCAS-BUCT

OPV 1-J Cell

Device ID: Y1

2:35 PM 10/3/2019

Spectrum: ASTM G173 global

Device temperature: 25.0 ± 0.5 °C

Device area: 0.048 cm^2 ± 0.5%

Irradiance: 1000.0 W/m^2

NATIONAL RENEWABLE ENERGY LABORATORY

OSMSS IV System

PV Performance Characterization Team

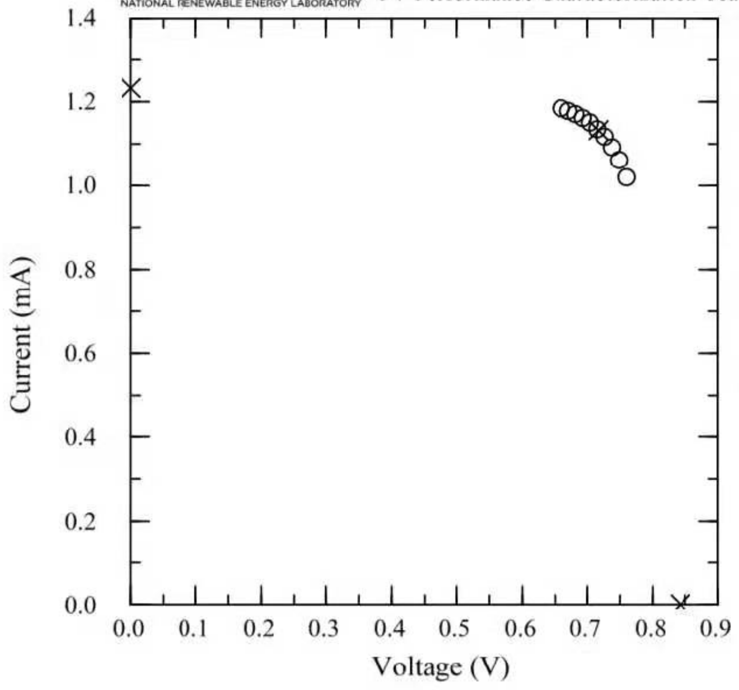

V_{oc} = 0.843 V ± 0.40%

I_{sc} = 1.245 mA ± 0.53%

J_{sc} = 25.704 mA/cm^2 ± 1.13%

Fill Factor = (77.5 ± 0.77)%

I_{max} = 1.132 mA ± 0.55%

V_{max} = 0.717 V ± 0.51%

P_{max} = 0.812 mW ± 0.67%

Efficiency = (16.77 ± 0.20)%

Asymptotic P_{max} scan

Elapsed time:631.5s.

Supplementary Figure 11. Certification report of the OSC based on PM6:Y6 with PDINN/Ag cathode from NREL.

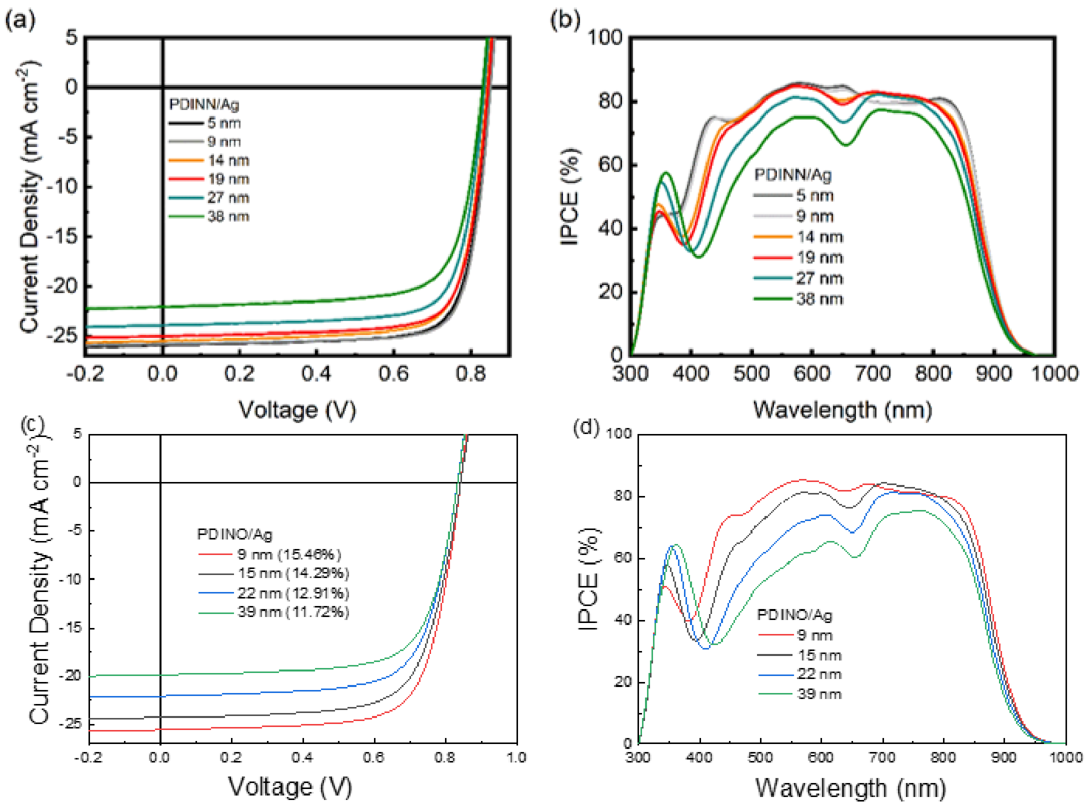

Supplementary Figure 12. (a) *J-V* curves and (b) IPCE spectra of the OSCs based on PM6:Y6 with PDINN CIM of different thickness; (c) *J-V* curves and (d) IPCE spectra of the devices with PDINO CIM of different thickness.

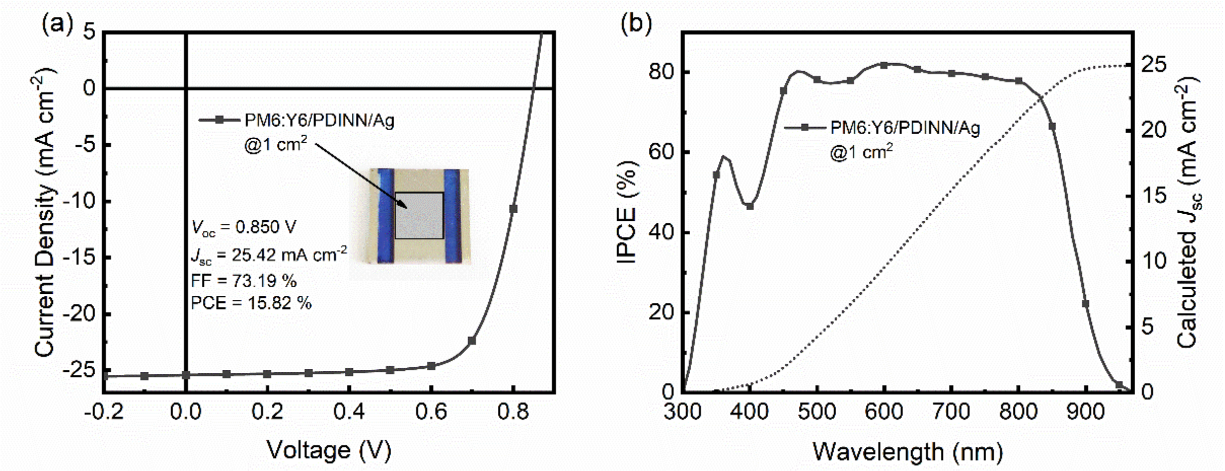

Supplementary Figure 13. (a) *J-V* curve and (b) *IPCE* spectrum of 1 cm^2 large area OSC with PDINN/Ag cathode.

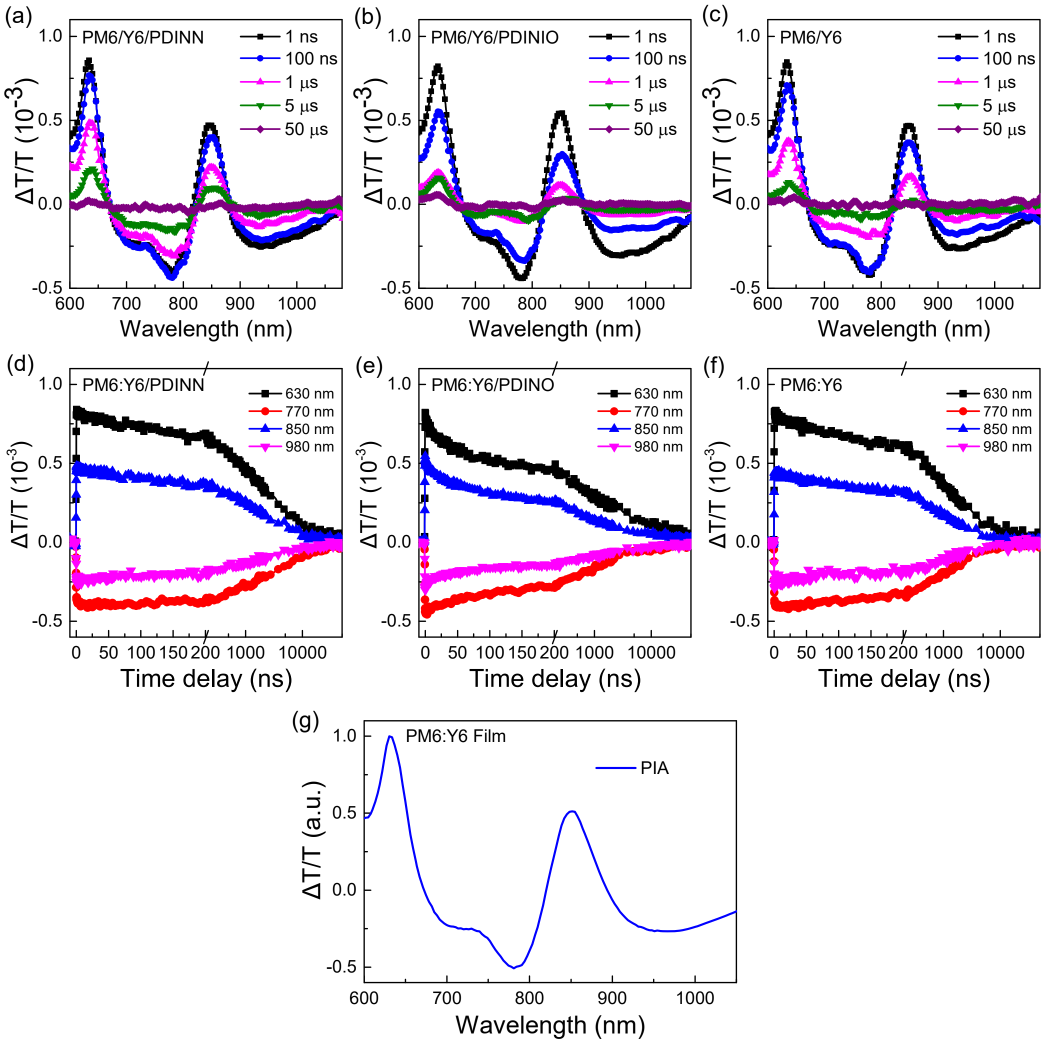

Supplementary Figure 14. Transient absorption spectra of (a) PM6:Y6/PDINN, (b) PM6:Y6/PDINO and (c) PM6:Y6 films recorded at different time delays with pump at 670 nm at a fluence of 1 μJ/cm^2. The dynamics probed at 630 nm, 770 nm, 850 nm and 980 nm for the three samples are shown in (d-e) respectively. (g) Photon-induced absorption (PIA) spectra of a PM6:Y6 film under weak cw excitation at 632. 8 nm. Upon weak continuous-wave excitation, the PIA is mainly contributed by the long-lived charge-separated state of free polarons. The PIA spectrum suggests that the excited-state absorption (ESA) features centered at 770 nm and 980 nm can be assigned to the charge separated state of charge polarons. Th slight difference between the dynamic curves probed at 770 nm and 980 nm is possibly due to different absorption cross sections of electron and hole polarons. The positive signals in the range of 600-670 nm and 820-880 nm are due to the ground state bleaching (GSB) of polymer donor and small molecule acceptor, respectively. The GSB of acceptor Y6 is slightly redshift to the absorption is due to the entanglement of excited-state absorption signal centered at 770 nm.

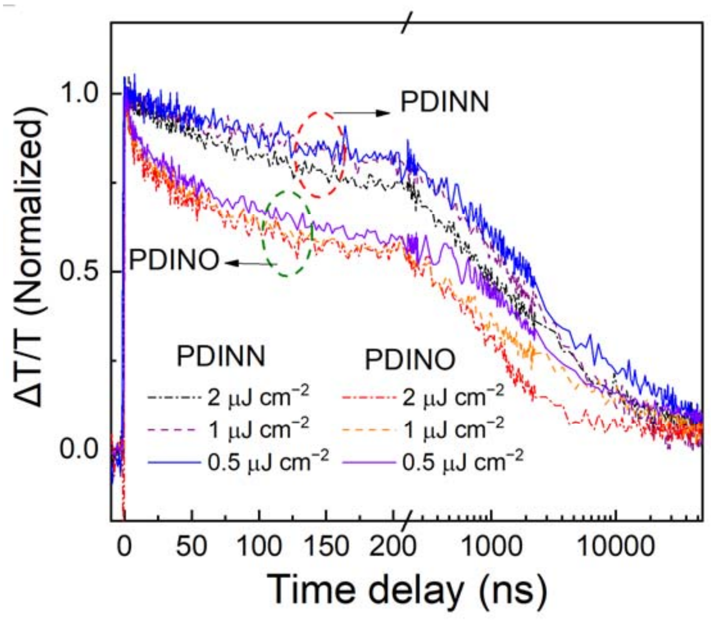

Supplementary Figure 15. Free charge dynamics of PM6:Y6/PDINN and PM6:Y6/PDINO films at different pump fluences. In all the pump conditions, free charge in PM6:Y6/PDINO decays faster than that in PM6:Y6/PDINN film.

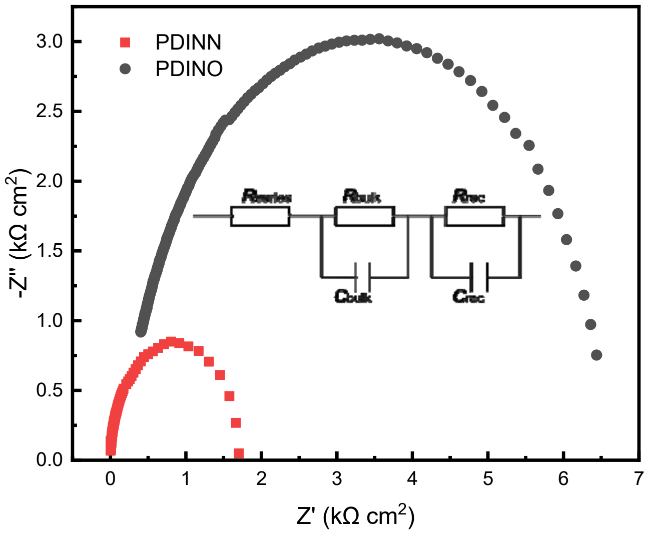

Supplementary Figure 16. Nyquist plots of the OSCs with PDINO or PDINN CIM at dark.

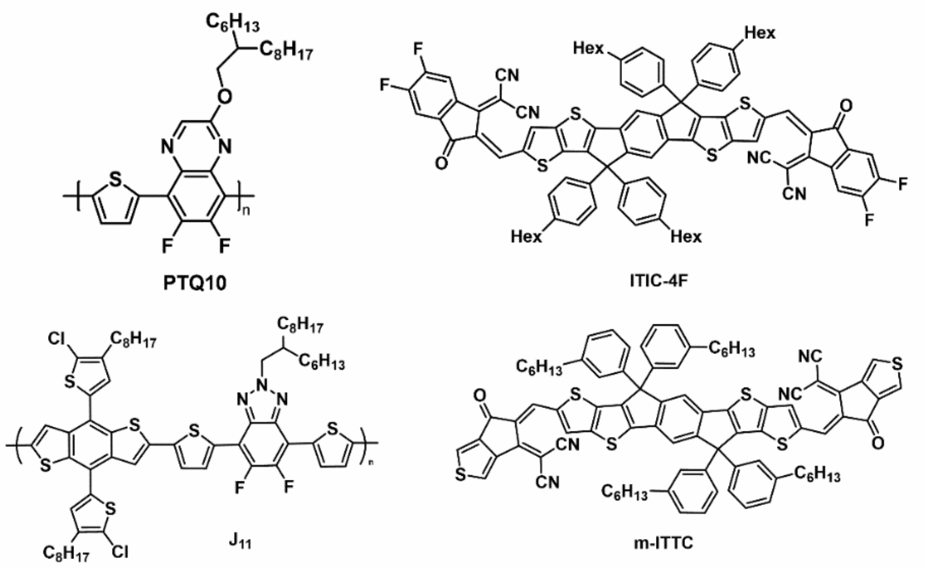

Supplementary Figure 17. Molecule structures of donors and acceptors used in the other OSCs.

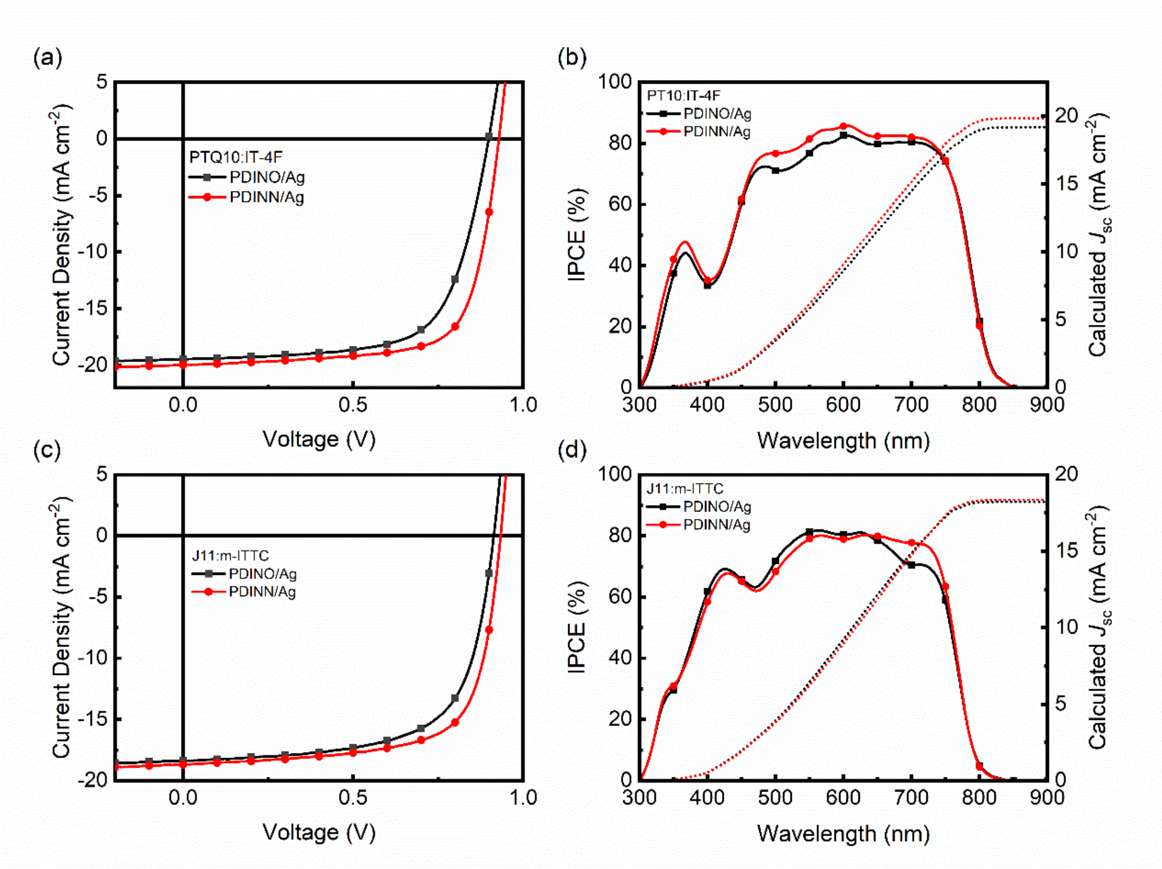

Supplementary Figure 18. (a), (c) *J-V* and (b), (d) IPCE spectra of the OSCs based on PDINN or PDINO CIMs.

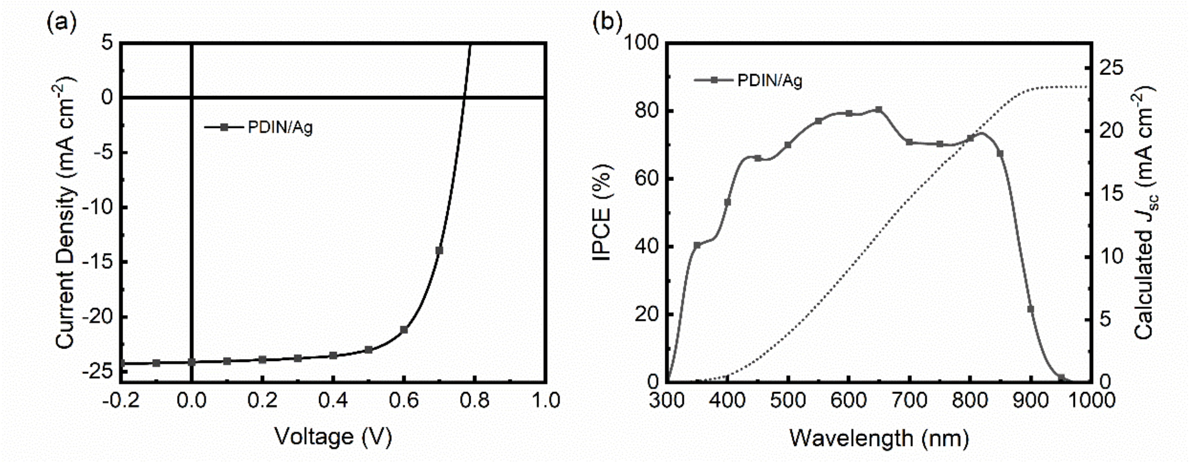

Supplementary Figure 19. (a) *J-V* and (b) IPCE spectrum of the OSC based on PM6:Y6 with PDIN/Ag as cathode.

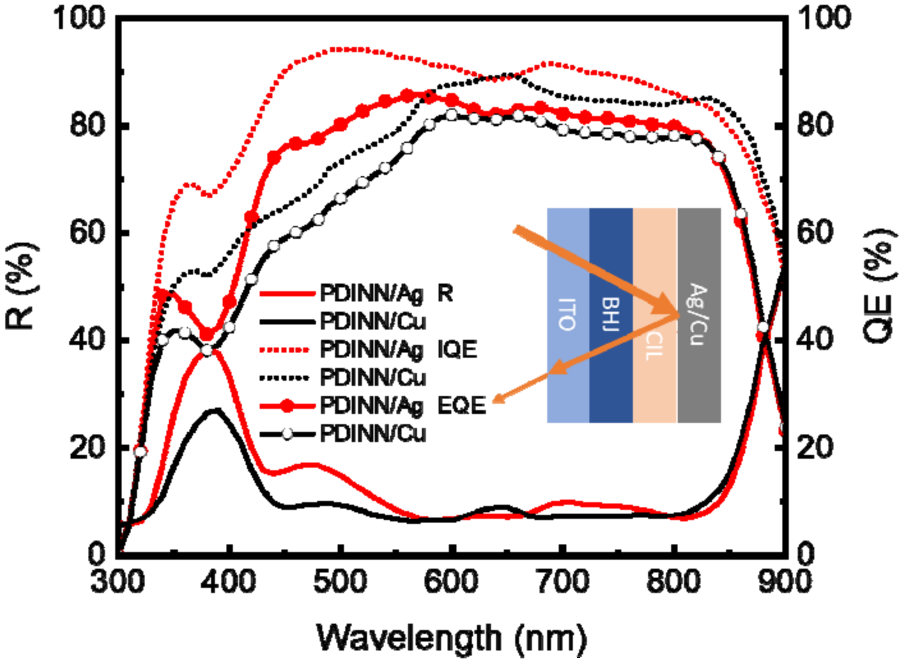

Supplementary Figure 20. Reflectivity, IQE and EQE values of the OSCs with PDINN/Ag or PDINN/Cu cathode. The inserted graph shows the light path in the OSC device and the thickness of the arrow lines represents the light intensity.

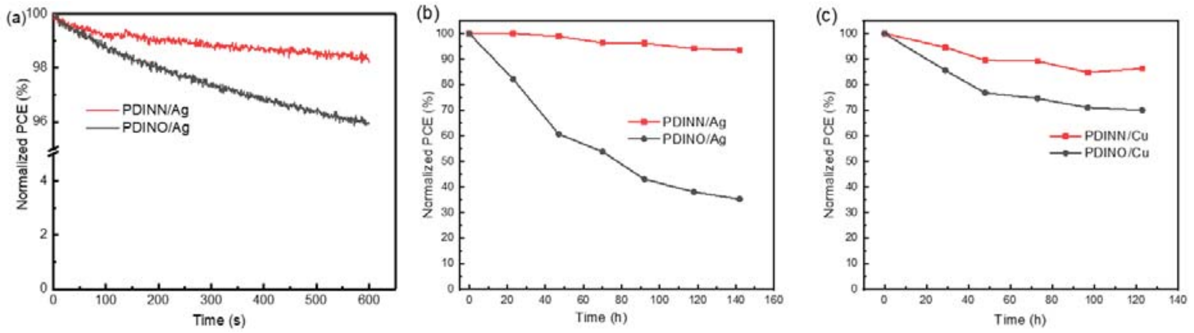

Supplementary Figure 21. (a) The steady-state efficiency of the devices operated at the maximum power voltage under continuous AM 1.5G illumination. The stability of the unencapsulated device with (b) Ag or (c) Cu as cathode, in air with the temperature of 25 °C and relative humidity of 40%.

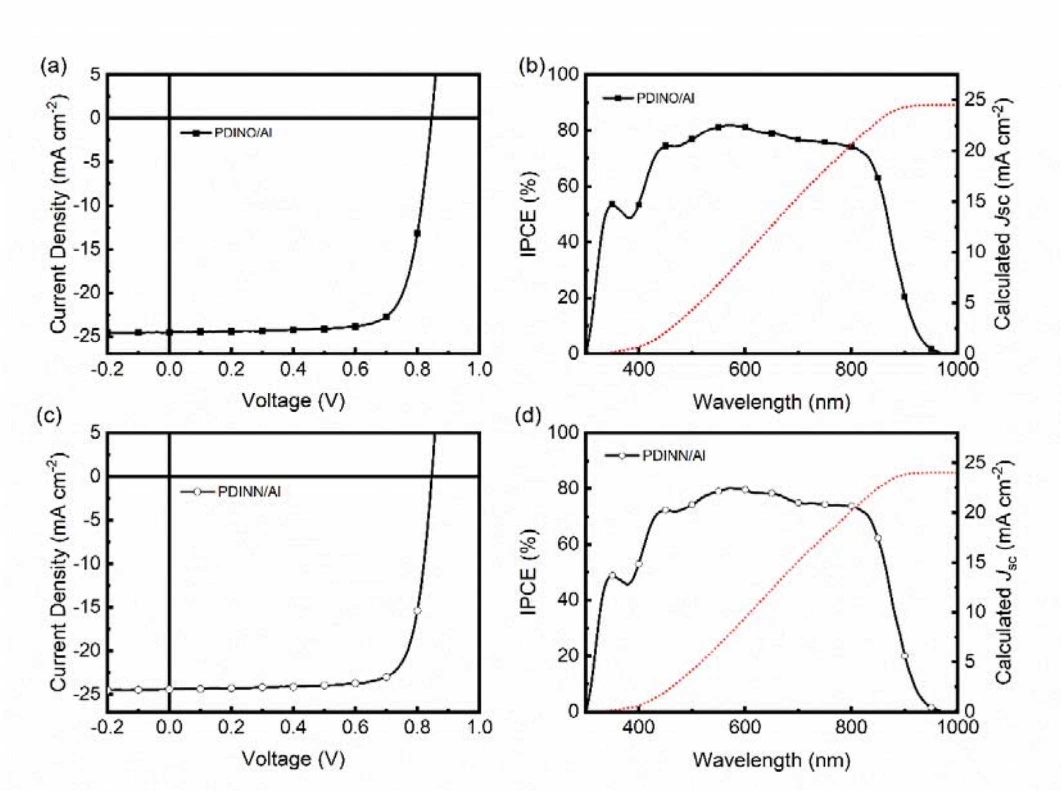

Supplementary Figure 22. (a)(c) *J-V* curves and (b)(d) IPCE spectra of the OSCs based on PM6:Y6/PDINO or PDINN with Al as cathode.

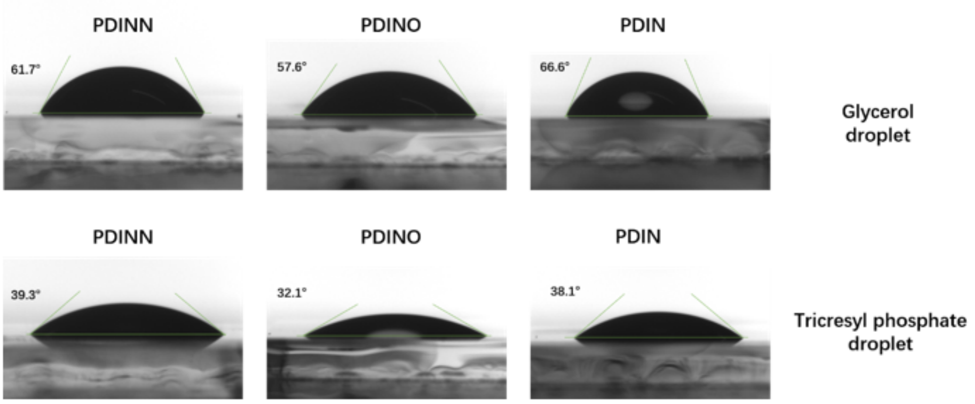

Surface	Glycerol Contact angle (°)	Tricresyl phosphate Contact angle (°)	Surface energy [mN m^{-1}]
PDINN	61.7°	39.3°	35.6
PDINO	57.6°	32.1°	38.6
PDIN	66.6°	38.1°	33.9

Supplementary Figure 23. Photographs of glycerol (top row) and tricresyl phosphate (bottom row) droplets in contact with the various surfaces. And summary of the measured contact angles, surface energies for the various surfaces.

SUPPLEMENTARY TABLES

Supplementary Table 1. The calculated chemical synthesis cost for PDINN with a Cg value of 1.64$/g

**Item**	**Chemicals**	**Quantity**	**Price (RMB)**	**Brand**	**Price of PDINN ($)**
Raw material	perylene-3,4,9,10-tetracarboxylic dianhydride	39.2 g	1188RMB/100g	J&K	110$/67.4g (1.64$ g^{-1})
	N,N-dimethyldipropylenetriamine	64.1 g	425RMB/100g	J&K	
Solvent	Methanol	300 mL	50 RMB/500 mL	Innochem	

Supplementary Table 2. Selected publications using PDINO and PDIN as CIMs.

CIM	Reference	Device structure
PDINO	*Joule* 2019, 3(4), 1140-1151	ITO/PEDOT:PSS /PM6: Y6/**PDINO**/Al
	Science, 2018, 361, 1094	ITO/PEDOT:PSS /PBDB-T:F-M/**PDINO**/Al
	National Science Review, 2020, DOI: 10.1093/nsr/nwz200	ITO/PEDOT:PSS / PBDB-TF: BTP-4Cl-12 /**PDINO**/Al
	Angew.Chem. Int.Ed. 2020, 59,2808	ITO/PEDOT:PSS / BTEC-2F:Y6 /**PDINO**/Al
	Nature Comm, 2016, **7**,13651	ITO/PEDOT:PSS/ J71:ITIC/**PDINO**/Al
	Energy Environ. Sci., 2019,12, 3400-	ITO/PEDOT:PSS/PTQ10:IDIC2F or IDIC/**PDINO**-G/Al
	Nature Comm 2018, 9, 743	ITO/PEDOT:PSS/PTQ10: IDIC/**PDINO**/Al
	Nature Comm 2019, 1, 519	ITO/PEDOT:PSS / PTQ10: MO-IDIC-2F /**PDINO**/Al
	J. Am. Chem. Soc., 2017, 139, 5085	ITO/PEDOT:PSS /H11:IDIC/**PDINO**/Al
	J. Am. Chem. Soc., 2016, 138, 4657	ITO/PEDOT:PSS/ J61: ITIC/**PDINO**/Al
	J. Am. Chem. Soc., 2016, 138, 15011	ITO/PEDOT:PSS/ J61: *m*-ITIC/**PDINO**/Al
	Angew. Chem. Int. Ed., 2017, 56, 13503	ITO/PEDOT:PSS/ PBDB‐T: PZ1 /**PDINO**/Al
	Angew. Chem. Int. Ed., 2018, 57, 4580	ITO/PEDOT:PSS/PBDB‐T: PNDI‐2T‐TR(5)/**PDINO**/Al
	Adv. Mater., 2015, 27, 1170	ITO/PEDOT:PSS/ PTB7‐Th : ITIC/**PDINO**/Al
	Adv. Mater. 2016, 28, 1884	ITO/PEDOT:PSS/ J51: N2200/**PDINO**/Al
	Adv. Mater., 2017, 29, 1604964	ITO/PEDOT:PSS/PBDB-T: FDICTF /**PDINO**/Al
	Adv. Mater. 2016, 28(25), 5112	ITO/NiO/Perovskite/PCBM/**PDINO**/Ag
	Adv. Mater., 2018, 30, 1704904	ITO/PEDOT:PSS/PBDB‐T: NCBDT/**PDINO**/Al
	Adv. Mater., 2018, 30, 1707508	ITO/PEDOT:PSS/PBDB-T:F-M/ZnO/n-PEDOT:PSS/PTB7-Th: NOBDYT/**PDINO**/Al
	Energy Environ. Sci., 2017,10, 1610	ITO/PEDOT:PSS/ J71: BT-IC /**PDINO**/Al
	Energy Environ. Sci., 2016,9, 3429	ITO/PEDOT:PSS/ J51: IDSe-T-IC /**PDINO**/Al
	Energy Environ. Sci., 2018,11, 2569	ITO/PEDOT:PSS/ PJ2: IDIC /**PDINO**/Al
	Adv. Funct. Mater. 2016, 26(25), 4543	ITO/PEDOT:PSS/ N(Ph‐2T‐DCN‐Et)3: PC71BM ITIC/ZnO/**PDINO**/AgNW
	Adv. Energy Mater., 2018, 8, 1702166	ITO/PEDOT:PSS/ PBDB‐TTn: *m*‐ITIC /**PDINO**/Al
	Adv. Energy Mater., 2018, 8, 1801618	ITO/PEDOT:PSS/PBDT‐T: IDTO‐T‐4/**PDINO**/Al
	Adv. Energy Mater., 2018, 8, 1701691	ITO/PEDOT:PSS/ PDOT: PC*71*BM: ITIC /**PDINO**/Al
	Adv. Energy Mater., 2019, 9, 1803976	ITO/PEDOT:PSS/ PBDB‐T:DTP‐IC‐4Ph /**PDINO**/Al

Adv. Energy Mater., 2017, 7, 1701125	ITO/PEDOT:PSS/ PTPTI－T70: m－ITIC /**PDINO**/Al
Adv. Energy Mater., 2017, 7, 1602215	ITO/PEDOT:PSS/ J51:PTB7－Th:ITIC/**PDINO**/Al
Adv. Energy Mater., 2018, 8, 1702870	ITO/PEDOT:PSS/PBDB－T: F–H, F–F, F–Cl, or F–Br /**PDINO**/Al
Adv. Electron. Mater., 2016, 2, 1600340	ITO/PEDOT:PSS/PIT2FBT:PC71BM/**PDIN**/Al,
Chem. Mater., 2017, 29, 8249	ITO/PEDOT:PSS/asy-PBDBTN:PC71BM or ITIC/**PDINO**/Al
Chem. Mater., 2016, 28, 5887	ITO/ PMOT5:PCBM /**PDINO**/Al
Chem. Mater., 2019, 31, 919	ITO/PEDOT:PSS/ PBB-T and ITIC-F /**PDINO**/Al
Chem.Mater.,2019 10.1021/acs.chemmater.9b01957	**ITO/PEDOT:PSS/ J52-TBF50: ITIC/PDINO/Al**
*Chem. Mater***.**, 2019, 31, 3025	**ITO/PEDOT:PSS/ PMTT56:IT-2F/PDINO/Al**
Chem. Mater., 2015, 27, 227	ITO/ PEDOT:PSS/$CH_3NH_3PbI_{3-x}Cl_x$/PCBM/**PDINO**/Ag
Chem. Mater., 2018, 30, 587	ITO/PEDOT:PSS/PBDB-T:NSTI/**PDINO**/Al
Nano Energy, 2019, 64, 103934	ITO/PEDOT:PSS /PBDB-T:IEICF-DMOT/**PDINO**/Al.
Nano Energy, 2016, 27, 430	ITO/PEDOT:PSS / J51:IDTIDSe-IC /**PDINO**/Al
Dyes and Pigments, 2018, 158, 445	ITO/PEDOT:PSS/DINTTDTS:IDT-C8/**PDINO**/
Polymer, 2019, 172, 391	ITO/PEDOT:PSS/PFBT-T:(PC71BM or IT-M)/**PDINO**/Al
Front.Chem., 2018, DOI: 10.3389/fchem.2018.00198	ITO/PEDOT: PSS/ PBDB-T:ITIC:PC71BM /**PDINO**/Al
Small, 2018, 14, 1704491	ITO/PEDOT:PSS/ PBTIBDTT－S: ITIC－F /**PDINO**/Al
Organic Electronics, 2016, 37, 396	ITO/ PAF-86 / MAPbI3/IPH /**PDINO**/Ag
Organic Electronics, 2018, 55, 106	ITO/PEDOT:PSS/ J41: ITIC /**PDINO**/Al
*J. Polym. Sci. Part A: Polym. Chem***.**, 2017, 55, 3679	ITO/PEDOT:PSS/ PTB7-Th:P(NDI2TOD-T2) /**PDINO**/Al
*Solar Energy***,** 2018, 173**,** 1107	ITO/PEDOT:PSS/ NDFP-CNCOO: PC71BM/**PDINO**/Al
*ACS Appl. Mater. Interfaces***,** 2018, 10**,** 41318	ITO/PEDOT:PSS/ HFAQx-T:N2200/**PDINO**/Al
*ACS Appl. Mater. Interfaces***,** 2018**,** 10**,** 23235	ITO/PEDOT:PSS/ P3: ITIC/**PDINO**/Al
*ACS Appl. Mater. Interfaces***,** 2016, 8, 15415	ITO/PEDOT:PSS/ PBDT-TS1: bis-PDI-T-EG /**PDINO**/Al
ACS Appl. Energy Mater., 2019, 2, 8, 6060	ITO/PEDOT:PSS/ PTB7-Th:PNDI-T5 or PTB7-Th:ITIC or PTB7-Th:$PC_{71}BM$ /**PDINO**/Ag
*Macromolecules***,** 2019, 52, 4359	ITO/PEDOT:PSS/ P3:PBDB-T:PNDI-2T-TR(5) /**PDINO**/Al
*Macromolecules***,** 2019, 52, 4464	ITO/PEDOT:PSS/ *PDCBT-Cl: ITIC-Th1* /**PDINO**/Al
*Macromolecules***,** 2018, 51, 2838	ITO/PEDOT:PSS/ *HFAQx-T:ITIC* **/PDINO**/Al
*Macromolecules***,** 2018, 51, 4032	ITO/PEDOT:PSS/ PBDB-T:PBDTO-T: IT-M /**PDINO**/Al
*Macromolecules***,** 2019, 52, 4776	ITO/PEDOT:PSS/ J76:*m*-ITIC /**PDINO**/Al
*Macromolecules***,** 2017, 50, 8928	ITO/PEDOT:PSS/TPhI-BDT or TffPhI-BDT: IDIC/**PDINO**/Al
Mater. Horiz., 2019 10.1039/C9MH00844F	ITO/PEDOT:PSS/ PBDB-T-SF：Y6：ITCT **/PDINO**/Al

	Nano Energy, 2016, 30, 312	ITO/PEDOT:PSS/ P*ff*Qx-T: ITIC /**PDINO**/Al
	Sci. China Mater., 2017, 60, 49	ITO/PEDOT:PSS/PTB7-Th:DCF2HT /**PDINO**/Al
	Dyes and Pigments, 2018, 148, 72	ITO/PEDOT:PSS/ PTBOQ-F: ITIC/**PDINO**/Al
	Dyes and Pigments, 2019, 169, 22	ITO/PEDOT:PSS/ FICBF-Se /**PDINO**/Al
	Dyes and Pigments, 2019, 163, 356	ITO/PEDOT:PSS/ PBDB-T:T-PDI /**PDINO**/Al
	Dyes and Pigments, 2019, 165, 18	ITO/PEDOT:PSS/PBDB-T:IBR/**PDINO**/Al.
	Sol. RRL, 2018, 2, 1800053	ITO/PEDOT:PSS/ PBDB - T: O - NTNC /**PDINO/Al**
	Optical Materials, 2017, 72, 147	ITO/PEDOT:PSS//NDFPX-DPP:$PC_{71}BM$/**PDINO**/Al
	Mol. Syst. Des. Eng., 2018,3, 103-112	ITO/PEDOT:PSS/ PMOT32: N2200 /**PDINO/Al**
	ACS Appl. Energy Mater., 2018, **1**, 2150	ITO/PEDOT:PSS/DRCN5T: IDIC8-F /**PDINO**/Al
	J. Mater. Chem. A, 2017,5, 17204-17210	ITO/PEDOT:PSS/ PBDB-T: NTIC /**PDINO**/Al
	Electrochimica Acta, 2016, 218, 263	ITO/CuSCN/CH3NH3PbI3/PCBM/**PDINO**/Al
**PDIN**	*J. Am. Chem. Soc*. 2017, 139, 4929	ITO/PEDOT:PSS/ PBDB-T:NFBDT /**PDIN**/Al
	ACS Nano, 2016, 10, 704	Al/**PDIN**/n-Si/PEDOT:PSS/HAT-CN/Ag
	Adv. Mater., 2016, 28, 3359	ITO/PEDOT:PSS/ PITFBT:PC71BM/**PDIN**/Al
	Energy Environ. Sci. 2015, 8, 610	ITO/PEDOT:PSS/ PTB7-Th: IEIC/**PDIN**/Al
	Organic Electronics, 2016, 35, 151	ITO/PEDOT:PSS/ F-DTS:R-PDI /**PDIN**/Ag
	ACS Appl. Mater. Interfaces 2018, 10,6, 5682-5692	ITO/PEDOT:PSS/ PTB7-Th:PC71BM /**PDIN**/BCP/Ag
	Dyes and Pigments, 2017, 139, 412	ITO/PEDOT:PSS/ PTB7-TH: PE-4DPPDCV /**PDIN**/Al
	J. Mater. Chem. A, 2016,4, 8724-8733	FTO/**PDIN**/MAPbI3−xClx/spiro-MeOTAD/Au
	J. Mater. Chem. A, 2017, 5, 12015	ITO/PEDOT:PSS/ PBIBD-O and PBIBD-T:PC71BM /**PDIN**/Al
	Org. Electron. 2018, 52, 200	ITO/PTAA/ perovskite/**PDIN**/Ag
	Polym. Chem., 2016,7, 2230-2238	ITO/PEDOT:PSS/PTB7-Th:Polymer acceptor/**PDIN**/Al
	J. Polym. Sci. Part A: Polym. Chem., 2018, 56, 105	ITO/PEDOT:PSS/ PTB7 - Th:PNDIT2Ptx /**PDIN**/Al
	ACS Appl. Mater. Interfaces, 2017, 9, 34, 28828	ITO/PEDOT:PSS/ PBDTSi-TA:ITIC/**PDIN/**BCP/Ag
	ACS Appl. Mater. Interfaces 2017,9,50,43688	ITO/ PAF-86 /CH3NH3PbIxCl3–x /**PDIN**/Ag

Supplementary Table 3. Photovoltaic Performance of the OSCs with different PDINN CIL thickness under the illumination of AM 1.5G,100 mW cm^{-2}.

Thickness [nm]	V_{oc} [V]	J_{sc} [mA cm^{-2}]	FF [%]	PCE [%]
5	0.850	25.92(25.89[a])	77.28	17.02
9	0.851	25.79(25.71[a])	78.64	17.25
14	0.842	25.48(25.21[a])	76.48	16.40
19	0.844	25.02(24.93[a])	77.00	16.25
27	0.834	23.90(23.59[a])	76.35	15.22
38	0.830	22.03(21.50[a])	74.09	13.55

[a] Calculated J_{sc} from IPCE

Supplementary Table 4. Photovoltaic Performance of the OSCs based on PDIN, PDINO and PDINN CIMs with different photoactive Layers under the illumination of AM 1.5G,100 mW cm^{-2}.

Active Layer	Devices	V_{oc} [V]	J_{sc} [mA cm^{-2}]	FF [%]	PCE [%]
PM6:Y6	PDINO/Al	0.843	24.55(24.52[a])	76.81	15.89
	PDINN/Al	0.846	24.41(24.04[a])	78.68	16.25
	PDIN/Ag	0.770	24.13(23.52[a])	68.52	12.74
PTQ10:IT-4F	PDINO/Al	0.914	19.21(19.14[a])	71.19	12.49
	PDINO/Ag	0.899	19.47(19.20[a])	67.60	11.83
	PDINN/Ag	0.929	19.97(19.86[a])	72.24	13.40
J11:m-ITTC	PDINO/Al[b]	0.920	18.62	70.9	12.15
	PDINO/Ag	0.914	18.37(18.24[a])	66.30	11.13
	PDINN/Ag	0.934	18.68(18.37[a])	70.02	12.21

[a] Calculated *J*sc from IPCE

[b] Result of high molecule weight J11 from Qiu, B. et al, A Simple Approach to Prepare Chlorinated Polymer Donors with Low-Lying HOMO Level for High Performance Polymer Solar Cells. *Chem. Mater.* **2019**, *31*(17), 6558-6567.

Supplementary Table 5. Photovoltaic Performance of the OSCs based on PM6:Y6/PDINO with different metal as cathode under the illumination of AM 1.5G,100 mW cm^{-2}.

Cathode	V_{oc} [V]	J_{sc} [mA cm^{-2}]	FF [%]	PCE[b] [%]
Al	0.843	24.55(24.52[a])	76.81	15.89
	0.844 ± 0.002	24.21 ± 0.38	76.47 ± 0.49	15.67 ± 0.28
Ag	0.821	25.58(24.94[a])	72.24	15.17
	0.816 ± 0.003	25.47 ± 0.25	71.80 ± 1.85	14.94 ± 0.42
Cu	0.814	24.22(24.02[a])	72.61	14.32
	0.814 ± 0.002	24.19 ± 0.53	71.42 ± 1.83	14.06 ± 0.46

[a] Calculated *J*sc from IPCE

[b] Average values with standard deviations were obtained from 10 devices.

Supplementary Table 6. Photovoltaic Performance of the OSCs based on PM6:Y6/PDINN with different metal as cathode under the illumination of AM 1.5G,100 mW cm^{-2}.

Cathode	V_{oc} [V]	J_{sc} [mA cm^{-2}]	FF [%]	PCE[b] [%]
Al	0.846	24.41(24.04[a])	78.68	16.25
	0.841 ± 0.004	24.21 ± 0.59	77.61 ± 0.99	15.80 ± 0.38
Ag	0.847	25.89(25.76 [a])	78.59	17.23
	0.845 ± 0.004	25.51 ± 0.28	77.84 ± 0.80	16.78 ± 0.33
Cu	0.844	24.28(24.04 [a])	78.80	16.15
	0.837 ± 0.004	24.13 ± 0.39	77.67 ± 0.88	15.69 ± 0.29

[a] Calculated J_{sc} from IPCE

[b] Average values with standard deviations were obtained from 10 devices.

Supplementary Table 7. The work of adhesion (W_a) and interfacial energy (γ_{ca}) for CIMs and active layer composite systems.

	W_a (mJ m^{-2})	γ_{ca} (mN m^{-1})
**PDINN/PM6[a]**	46.13	5.17
**PDINO/PM6[a]**	48.02	6.28
**PDINN/Y6[a]**	43.83	6.07
**PDINO/Y6[a]**	45.66	7.24

Supplementary Methods.

Owen Method for calculating the surface energy

The Owen's method [1] is often used to calculate the surface energy:

$$\gamma_s = \gamma_s^D + \gamma_s^P, \gamma_l = \gamma_l^D + \gamma_l^P \tag{1}$$

where γ_s is composed of the dispersion force γ_s^D and polarity force γ_s^P. γ_l is surface energy of the liquid and consists of a dispersion force γ_s^D and polarity force γ_s^P.

We can know the surface energies γ_l^D and γ_l^P of the testing liquid and its contact angle on solid film. And according to the formula:

$$\gamma_l(1 + cos\theta) = 2(\gamma_s^D\gamma_l^D)^{1/2} + 2(\gamma_s^P\gamma_l^P)^{1/2} \tag{2}$$

We need two known testing liquids to determine γ_s^D and γ_s^P.

$$\gamma_{l1}(1 + cos\theta) = 2(\gamma_s^D\gamma_{l1}^D)^{1/2} + 2(\gamma_s^P\gamma_{l1}^P)^{1/2} \tag{3}$$

$$\gamma_{l2}(1 + cos\theta) = 2(\gamma_s^D\gamma_{l2}^D)^{1/2} + 2(\gamma_s^P\gamma_{l2}^P)^{1/2} \tag{4}$$

Finally, γ_s can be determined by $\gamma_s = \gamma_s^D + \gamma_s^P$.

The work of adhesion (W_a) and the interfacial energy (γ_{ca}) are calculated according to the reference.[2] The values of surface energy of PM6 and Y6 are from the literature.[3]

W_a is the work required to disjoin a unit area of the solid-solid interface by creating a unit area of solid–vacuum interface, and defined by the equation:

$$W_a = \gamma_c + \gamma_a - \gamma_{ca} \tag{5}$$

where γ_c is the CIM surface energy, γ_a is the active layer surface energy and γ_{ca} is interfacial energy.

As the surface energies of the CIMs and the active layer are described following the Owens–Wendt approach, the work of adhesion can be evaluated using the following equation:

$$W_a = 2\left(\sqrt{\gamma_c^D\gamma_a^D} + \sqrt{\gamma_c^P\gamma_a^P}\right) \tag{6}$$

Supplementary References

1. Owens, D.K. & Wendt, R.C. Estimation of the surface free energy of polymers. *J. Appl. Polym. Sci.* **13**, 1741-1747 (1969).
2. Tran, L.Q.N., Fuentes, C.A., Dupont-Gillain, C., Van Vuure, A.W. & Verpoest, I. Understanding the interfacial compatibility and adhesion of natural coir fibre thermoplastic composites. *Compos. Sci. Technol.* **80**, 23-30 (2013).
3. Xu, Y.*, et al.* Tuning the Hybridization of Local Exciton and Charge-Transfer States in Highly Efficient Organic Photovoltaic Cells. *Angew. Chem. Int. Ed.*, DOI: 10.1002/anie.201915030 (2020).
