# Supplementary material for: Cathode engineering with perylene-diimide interlayer enabling over 17% efficiency single-junction organic solar cells
Source: Nat Commun. 2020 Jun 1;11:2726. doi: 10.1038/s41467-020-16509-w (PMC7264349; doi:10.1038/s41467-020-16509-w)
Supplement: Supplementary file 3 — Solar Cells Reporting Summary [file 41467_2020_16509_MOESM3_ESM.pdf]

nature research

Corresponding author(s): Zhi-Guo Zhang; Yongfang Li

Solar Cells Reporting Summary

Nature Research wishes to improve the reproducibility of the work that we publish. This form is intended for publication with all accepted papers reporting the characterization of photovoltaic devices and provides structure for consistency and transparency in reporting. Some list items might not apply to an individual manuscript, but all fields must be completed for clarity.

For further information on Nature Research policies, including our data availability policy, see Authors & Referees.

Experimental design

Please check: are the following details reported in the manuscript?

1. Dimensions

Item	Yes	No	Response
Area of the tested solar cells	☒	☐	Area of the tested solar cells is provided in Methods, section"Device fabrication and characterization of the OSCs"
Method used to determine the device area	☒	☐	The method is provided in Methods, section"Device fabrication and characterization of the OSCs"

2. Current-voltage characterization

Item	Yes	No	Response
Current density-voltage (J-V) plots in both forward and backward direction	☐	☒	Only the plots in forward direction was supplied.
Voltage scan conditions<br>*For instance: scan direction, speed, dwell times*	☒	☐	The scan condition is provided in Methods, section"Device fabrication and characterization of the OSCs"
Test environment<br>*For instance: characterization temperature, in air or in glove box*	☒	☐	The environment is provided in Methods, section"Device fabrication and characterization of the OSCs"
Protocol for preconditioning of the device before its characterization	☐	☒	No preconditioning protocol.
Stability of the J-V characteristic<br>*Verified with time evolution of the maximum power point or with the photocurrent at maximum power point; see ref. 7 for details.*	☒	☐	We measured the steady output efficiencies of the PDINN- and PDINO-based devices with voltages at their maximum power points under AM 1.5G illumination, as shown in Supplementary Figure 21a.

3. Hysteresis or any other unusual behaviour

Item	Yes	No	Response
Description of the unusual behaviour observed during the characterization	☐	☒	No hysteresis was observed in our device.
Related experimental data	☐	☒	No.

4. Efficiency

Item	Yes	No	Response
External quantum efficiency (EQE) or incident photons to current efficiency (IPCE)	☒	☐	IPCE curve is shown in Figure 2b and Figure 4c.
A comparison between the integrated response under the standard reference spectrum and the response measure under the simulator	☒	☐	Relative information is provided in Table 2, Supplementary Table 5 and 6.
For tandem solar cells, the bias illumination and bias voltage used for each subcell	☐	☒	Our cells were single solar cells.

5. Calibration

Item	Yes	No	Response
Light source and reference cell or sensor used for the characterization	☒	☐	Relative information is provided in Methods, section"Device fabrication and characterization of the OSCs"
Confirmation that the reference cell was calibrated and certified	☒	☐	Relative information is provided in Methods, section"Device fabrication and characterization of the OSCs"

Calculation of spectral mismatch between the reference cell and the devices under test

- [ ] Yes
- [x] No

Spectral mismatch factor was not considered.

6. Mask/aperture

Size of the mask/aperture used during testing

- [ ] Yes
- [x] No

We didn't use masks during testing in the lab, but a device with a mask of 0.048 cm2 was certified by National Renewable Energy Laboratory (NREL), USA.

Variation of the measured short-circuit current density with the mask/aperture area

- [x] Yes
- [ ] No

The masked and unmasked test results are listed in Table 2.

7. Performance certification

Identity of the independent certification laboratory that confirmed the photovoltaic performance

- [x] Yes
- [ ] No

The photovoltaic performance of our device was Certified by National Renewable Energy Laboratory (NREL), USA.

A copy of any certificate(s)
Provide in Supplementary Information

- [x] Yes
- [ ] No

Relative information is provided in supplementary information, Supplementary Figure 11.

8. Statistics

Number of solar cells tested

- [x] Yes
- [ ] No

Number of solar cells tested is provided in Table 2, Supplementary Table 5 and 6.

Statistical analysis of the device performance

- [x] Yes
- [ ] No

Statistical results of the devices are listed in Table 2, Supplementary Table 5 and 6.

9. Long-term stability analysis

Type of analysis, bias conditions and environmental conditions
For instance: illumination type, temperature, atmosphere humidity, encapsulation method, preconditioning temperature

- [x] Yes
- [ ] No

Long-term stability analysis can be found in the section “Device Stability and Effect of Cathodes on Device Performance"
